# Special Issue “Molecular Detection and Typing of Viruses”

**DOI:** 10.3390/diagnostics11112031

**Published:** 2021-11-03

**Authors:** Ilka Engelmann

**Affiliations:** Laboratoire de Virologie, Université Lille, CHU Lille, ULR3610, F-59000 Lille, France; Ilka.ENGELMANN@CHRU-LILLE.FR; Tel.: +33-320-444-880

I thank all authors, reviewers and the editorial staff who contributed to this special issue. The articles published in this special issue highlight how molecular biology has revolutionized virology. Molecular biology is nowadays largely used for virus detection in routine diagnosis, for virus typing and phylogenetic analysis. It is also useful for the discovery of new viruses that may be pathogens or members of our healthy virome. An example of how central the role of molecular biology has become to virology, is a recently discovered virus that became a public health concern, the severe acute respiratory syndrome coronavirus 2 (SARS-CoV-2). This virus was first identified by next generation sequencing [1]. One article in this special issue addresses the challenges of discovering new viruses by using next generation sequencing [2]. Following the spread of SARS-CoV-2 that rapidly caused a pandemic, it was then searched for and detected massively by reverse-transcription polymerase chain reaction (RT PCR). At first, laboratory developed assays became available, the first ones already in January 2020, only two weeks after the announcement of the novel coronavirus as causative pathogen of pneumonia [3]. Then, a wealth of commercial assays were developed. A study published in this special issue [4] applied RT PCR to different specimen types. This allowed to characterize spatio-temporal virus load dynamics of this new virus and shed some light on the pathophysiology of the infection, termed COVID-19 [4]. Molecular assays for detection of SARS-CoV-2 variants have next been developed to screen for variants of concern, with one example being published in this special issue [5]. Lately, next generation sequencing has become widely used to track down new variants and follow the evolution of this new virus [6]. Two other reports in this special issue describe the use of molecular biology techniques applied to phylogenetic studies and typing of hepatitis C and influenza A viruses, respectively [7,8]. Last but not least, molecular biology also is the basis of most of the currently available vaccines against SARS-CoV-2, that are either RNA based or viral vector based [9]. Taken together, this shows that we can no longer conceive virology without molecular biology.
